# Molecular Diagnostic Tests for Microsporidia

**DOI:** 10.1155/2009/926521

**Published:** 2009-08-02

**Authors:** Kaya Ghosh, Louis M. Weiss

**Affiliations:** ^1^Department of Pathology, Albert Einstein College of Medicine, Bronx, NY 10461, USA; ^2^Department of Biological Sciences, Rutgers University, Newark, NY 07102, USA; ^3^Department of Medicine, Albert Einstein College of Medicine, Bronx, NY 10461, USA

## Abstract

The Microsporidia are a ubiquitous group of eukaryotic obligate intracellular parasites which were recognized over 100 years ago with the description of *Nosema bombycis*, a parasite of silkworms. It is now appreciated that these organisms are related to the Fungi. Microsporidia infect all major animal groups most often as gastrointestinal pathogens; however they have been reported from every tissue and organ, and their spores are common in environmental sources such as ditch water. Several different genera of these organisms infect humans, but the majority of infections are due to either *Enterocytozoon bieneusi* or *Encephalitozoon* species. These pathogens can be difficult to diagnose, but significant progress has been made in the last decade in the development of molecular diagnostic reagents for these organisms. This report reviews the molecular diagnostic tests that have been described for the identification of the microsporidia that infect humans.

## 1. Introduction

 The Microsporidia are a phylum of over 1200 species representing at least 150 genera [1, 2]. Since the mid-1980s, these organisms have increasingly been implicated as agents of human disease, especially in their capacity as opportunistic pathogens in patients with HIV infection [2, 3] and other immunosuppressed individuals, such as those with organ transplantation or chemotherapy recipients [4]. To date, fourteen species in eight genera have been found to infect humans [5]. In HIV-positive patients, the most common clinical manifestation is chronic diarrhea and wasting due to enteric infection, but the spectrum of disease due to these pathogens is broad and includes hepatitis, peritonitis, keratoconjunctivits, sinusitis, bronchitis, pneumonia, cystitis, nephritis, myositis, encephalitis, and other cerebral infections [4]. In addition, microsporidia have also been reported to be etiologic in isolated case reports of urethritis, prostatic abscess, tongue ulcer, bone infection, and cutaneous infection [4]. There is an increasing appreciation that these organisms can also cause gastrointestinal and ocular infections in apparently immunocompetent individuals. Serosurveys [6, 7] suggest that microsporidiosis is common, but usually self-limiting or asymptomatic in the general population. While transmission routes have not been specifically documented in epidemiologic studies, there is evidence that infections can occur by multiple routes (enumerated in [2]) including waterborne, respiratory, sexual, congenital, zoonotic transmission, and in ocular infection by traumatic inoculation into the cornea.

All microsporidia produce an environmentally resistant spore which is capable of extruding its coiled, internal polar filament (i.e., polar tube) thereby inoculating its contents into a nearby host cell. Unique in structure and function, identification of the polar filament is diagnostic for the phylum. Due to the small size of the organisms, for example, several of the human-infecting species measure 1–2 *μ*m [4], diagnosis of microsporidiosis has traditionally relied on transmission electron microscopy (TEM) to identify the polar filament and other phylum- and species-specific ultrastructural characters. Although it remains the gold standard, TEM is labor-intensive and time-consuming, requiring expensive equipment, significant specialized expertise, and a dedicated histological staff working over the course of several days. It is also relatively insensitive, due to the small amount of tissue that can be examined and the lack of signal amplification. Light microscopy-based methods have also been developed and are faster and typically more sensitive than TEM, but they still require experienced pathologists for successful interpretation. These methods include routine histological stains such as the modified trichrome stain which is used alone or in combination with other stains such as Gram or Warthin-Starry silver [1]. Although these methods are more convenient than TEM for detecting microsporidia in body fluids and tissues, the internal polar filament is not easily identified using these techniques. Rather, diagnosis hinges mostly on the detection of the thick spore wall which is birefringent and provides selective staining characteristics with the modified trichrome stain. Chemofluorescent brighteners (e.g., Calcofluor White, Uvitex 2B, Fungifluor) have been used to target the chitin within the spore wall. While sensitive, the potential for cross reactivity with Fungi and artifactual material exists, especially in stool specimens. Thus, it has been recommended that chemofluorescent brighteners should be used in combination with traditional histological stains, to provide better sensitivity and specificity when examining stool specimens. However, even the best possible tissue preparation and staining for light microscopy rarely enables a microsporidian species-specific diagnosis. This is a critical shortcoming in light of the need for different treatments for the various microsporidian species that infect humans [8].

While TEM evidence of the polar filament or other ultrastructural features unique to the phylum is considered incontrovertible proof of microsporidiosis, a more specific diagnosis is not always possible on the basis of morphology alone. Especially in the case of closely related species, distinguishing characteristics may arise in only certain developmental stages of the organism, all of which may not be present in a particular clinical sample. While in vitro culture is conceivable as a tool to aid in diagnosis for several human-infecting species, culture methods are laborious, subject to contamination, and usually impractical; moreover, for *Enterocytozoon bieneusi*, the most common microsporidium found in humans, no in vitro culture system exists [9]. Thus, there exists a need for faster, more specific, and more accessible approaches to diagnosis in both clinical specimens and environmental samples. 

Over the past decade or so, molecular biology-based procedures have been increasingly used in clinical settings for the diagnosis and characterization of microbial pathogens. These procedures are designed to detect either a nucleic acid sequence or antigen specific to the pathogen. Compared to traditional microscopy- or culture-based methods, molecular methods can offer the following potential advantages: increased sensitivity, by virtue of amplification of signal; greater specificity, when appropriate detection probes are employed; faster time-to-result; and greater ease of interpretation by nonspecialists [10]. While clinical laboratories still primarily rely on microscopy-based methods for the diagnosis of microsporidia, over the past fifteen years significant effort has been directed to the development of molecular methods in research laboratories. This article will review the progress toward molecular diagnostics of these emerging pathogens.

## 2. Nucleic Acid-Based Detection Methods

 Nucleic acid-based detection methods utilize synthetic DNA molecules that are specific and complementary to a sequence in the DNA of the pathogen. The earliest methods employed labeled probes which hybridized to pathogen DNA and emitted a detectable (e.g., fluorescent) signal. Such DNA probe technologies are still in use today, although they have been largely supplanted by methods that amplify the target sequence; of these methods the most commonly utilized is the polymerase chain reaction (PCR) (for a historical perspective, see [11]). In PCR, the target pathogen DNA is bound by a specifically designed set of primers and copied over and over again in the presence of free nucleotides by a themostable polymerase enzyme. The amplification of the target pathogen DNA (i.e., amplicon) confers two advantages: improved detection sensitivity relative to probe-based methods and facilitation of downstream analyses (e.g., restriction analysis, sequencing) of the amplicon. 

Techniques for sample preparation for the molecular diagnosis of microsporidia have been reviewed in detail in Weiss and Vossbrinck [12]. The technique used to extract DNA for amplification can significantly affect the sensitivity of a PCR diagnostic technique. Nucleic acids may be extracted from clinical samples such as tissue biopsies, corneal scrapings, duodenal aspirations, and urine specimens as well as in vitro cultures with commercial DNA extraction kits (e.g., those manufactured by Qiagen, Santa Clara, Calif, Usa, or Promega, Madison, Wis, Usa) or by routine procedures such as proteinase K digestion followed by phenol-chloroform extraction and ethanol precipitation [13]. DNA may also be isolated from paraffin-embedded material by standard methods [14] or with commercial kits such as DexPAT (Takera Biochemical, Berkeley Calif, Usa). DNA has been successfully amplified from modified trichrome-stained [15] and decades-old Giemsa-stained [16] microscope slides by scraping the material off of the slides followed by mechanical disruption of the microsporidia (using glass beads) and subsequent DNA extraction using standard techniques. 

The isolation and amplification of DNA from stool samples is more challenging, generally requiring mechanical disruption and/or harsh extraction conditions. Successful reported methods include subjection to 0.5% sodium hypochlorite [17], chitinase [17], lyticase [18], guanidine thiocyanate [19–21], 10% formalin or M potassium hydroxide [17, 22, 23], dithiothreitol [22], hexadecyltrimethylammoniuim bromide [24], or boiling the samples (Ombrouck et al. [25]. Stool samples frequently contain inhibitors of polymerase enzymes [26]. If they are not removed by the above methods, dilution of the samples (Ombrouck et al.,[27]) or guanidine thiocyanate treatment [19] may be warranted. An extraction-free template preparation method for stool samples has also been developed [28] using FTA filters impregnated with denaturants, chelating agents, and free-radical traps, which apparently causes most cells to lyse on contact and enables debris and other inhibitory factors to be washed away from the DNA trapped on the filter. In one study this FTA filter method allowed the detection of 800 microsporidian spores per milliliter of stool by a PCR technique (Subrungruang et al., [29]).

In order to apply PCR-based diagnostics to a pathogen, some genetic sequence information must be known in advance. The human-infecting microsporidia are a diverse group of “emerging” pathogens, and the available genetic information on these organisms is limited but ever-increasing. For the majority of the microsporidia GenBank sequence data on their rRNA genes is the only genetic information available. The number of microsporidian genes deposited in GenBank has grown from less than 200 in 1999 (surveyed in [12]) to almost 6000 today. Approximately two-thousand of these are the genome sequence-related predicted genes proposed by Katinka et al. [30] in their landmark genomic sequencing of the human pathogen *Encephalitzoon cuniculi*, the first and to-date only microsporidium genome to be completely sequenced. For the closely related species *Encephalitozoon hellem*, there are 75 entries. Recently, a genomic survey of the essentially noncultivatable pathogen *Enterocytozoon bieneusi *resulted in the addition of another three-thousand hypothetical genes, some of which are homologs to the genes identified in *Enc. cuniculi* [31]. For the other eleven human-infecting species of microsporidia, between zero and a few dozen genes have been deposited in GenBank. 

Due to the availability of sequence information as well as the presence of conserved and variable regions within the rRNA genes, PCR-based methods have typically utilized primers to this gene for the characterization of the microsporidia. The first such report of the use of conserved rRNA primers was of that of the cloning of the small subunit (SSU) rDNA of *Vairimorpha necatrix*, a pathogen of agricultural pests [32]. Primers complementary to conserved sequences within this gene were used to amplify and subsequently obtain sequence data on the rRNA gene of several human-infecting microsporidia, including *Enc*. *cuniculi*, *Enc*. *hellem*, *Enc*. *intestinalis*, *Ent*. *bieneusi*, and *Vittaforma corneae* (reviewed in [33, 34]). These rRNA genes have been reported by Katinka et al. [30] to be present in more than twenty copies in the *Enc*. *cuniculi* genome, and therefore, provide an increase in sensitivity (over single copy genes) for use in diagnostic PCR tests. 

Diagnostic studies using primers to the various rRNA genes of microsporidia have been reviewed by Weiss and Vossbrinck [12] and Franzen and Muller [35]. The sequences of many of the primer pairs used for the amplification of various microsporidia, along with the recommended annealing temperatures for PCR and the expected amplicon size, are compiled in Table 1 (adapted from [12]). Some of these primers are species-specific whereas others are more general primer sets that amplify all of the Encephalitozoonidae. For some of these primer sets downstream restriction analysis, wherein the amplicon is digested into smaller pieces by specific restriction enzymes, is required for species specific diagnoses. 

PCR has also been useful for the identification of the previously unknown microsporidia in human and veterinary infections. Using phylogenetically conserved primers amplifying the small subunit (SSU), large subunit (LSU), and intergenic spacer (IGS) regions, it has been possible to clone and then sequence portions of the rRNA gene of uncharacterized microsporidia from biopsy specimens (Table 2, adapted from [12]). These rRNA sequence data can then be used for phylogenetic analysis using BLAST and similar in silico programs comparing the unknown sequence to the rRNA sequences on various microsporidia available in GenBank. The primers in Table 2 form the basis of a “molecular toolbox” which allows the cloning of rRNA genes from novel species or strains of microsporidia. The primer pairs V1(18f)::1492r and 530f::580r are considered “universal” in that they are usually successful in amplifying unknown rRNA genes for novel species or strains of microsporidia ([36], also see [12]). 

Several investigators have published diagnostic procedures for microsporidia which use real-time PCR [33, 34, 53, 54, 56]. Real-time PCR, which detects accumulating amplicons in real time via interacting either fluorescent dyes or fluorescence-labelled probes, has the advantage of being quantitative over a broad dynamic range. In addition, it typically employs a multiwell format and dispenses with postamplification processing of the sample, which increases throughput and reduces the risk of contamination inherent in PCR [57]. Hester et al. [53] used a probe specific for the small subunit rRNA of the genus *Encephalitozoon* and species-specific primers for *Enc. cuniculi, Enc. hellem,* and *Enc. intestinalis.* While their method was validated only for purified microsporidian DNA, it could be adapted for clinical samples. Another study utilized pan-*Encephalitozoon* primers specific to small subunit rRNA and a guanidine thiocyanate-based extraction system designed for an automated workstation to detect *Enc*. *cuniculi*, *Enc*. *hellem*, and *Enc*. *intestinalis* from stool specimens [56]. The assay was sensitive (detection limit between 10^2^ to 10^3^ spores/mL) and reflective of infection intensity (linear range was between 10^3^ and 10^7^ spores/mL). In addition, melting curve analyses of the amplicons readily allowed differentiation of the three *Enc*. species., which is useful for multiple or unknown infections. In 2003, a real-time PCR assay using primers for *Enc*. *intestinalis* small subunit rRNA was used to detect this pathogen from known clinical samples, including stools, urine, tissue biopsies, bronchopulmonary specimens, and blood [33, 34]. Using control reference spores, the detection limit was estimated to be 20 spores per milliliter, which was sufficient to detect a relatively low-intensity blood infection suggesting that an infection was disseminated [33, 34]. Finally, a multiplex real-time PCR assay has also been reported to simultaneously detect *Ent*. *bieneusi*, *Enc*. *cuniculi*, *Enc*. *hellem*, and *Enc*. *intestinalis* from both fresh and formalin-fixed stool with primers for the intergenic region and small subunit rRNA of *Ent*. *bieneusi* and *Encephalitozoon* species., respectively [54]. *Ent*. *bieneusi* was detected in 30 of 33 known microsporidia-positive samples. The study included a range of negative and positive controls to verify the assay specificity and guard against false negatives due to inhibitors potentially present in stool or to the presence of extraneous DNA, respectively. 

A few studies have also utilized fluorescent *in situ* hybridization (FISH-) based methods to detect microsporidia. Essentially, FISH technology utilizes a fluorescence-labeled probe that binds to complementary nucleic acid (DNA or RNA) in the specimen [10]. In contrast to PCR, general morphological and spatial information regarding probe-binding in the specimen may be retained because the procedure is performed *in situ*. FISH has been used with probes against the small subunit or intergenic regions of microsporidia rRNA to detect *Ent*. *bieneusi* and *Enc*. *hellem* [24, 51, 52]. These methods were used successfully with archived formalin-fixed, paraffin-embedded (FFPE) clinical samples and detected either more microsporidia-positive samples or more infected cells within samples than traditional histochemical staining. In the case of *Ent. bieneusi*, characteristic staining of parasites in a supranuclear location within jejunal biopsy epithelial cells [24] and staining of developmental forms [52] contributed to the certainty of diagnosis. Although FISH is an attractive procedure due to the multifaceted information, it can provide two factors seriously hamper its potential for general use in clinical diagnostic settings. Firstly, it is rather laborious and technically challenging, requiring deparaffinization, dehydration, and rehydration (of FFPE samples), digestion by proteinases to make the nucleic acid accessible to the probe, labeling and overnight hybridization of the probe, blocking, counterstaining, and many wash steps prior to viewing with an epifluorescence-equipped microscope. Secondly, it is less sensitive than PCR by orders of magnitude due to the lack of amplification of original signal (i.e., the nucleic acid target). Nonetheless, it may prove particularly useful for environmental samples wherein discrimination of live versus dead organisms is important, which can be afforded by designing probes for the less-stable RNA rather than DNA (discussed in [51]).

Of interest is the report of the development of an oligonucleotide microarray to simultaneously detect *Ent*. *bieneusi*, *Enc*. *cuniculi*, *Enc*. *hellem*, and *Enc. intestinalis * from clinical samples [55]. Such microarrays were originally developed for genomewide expression analysis but have recently been applied to molecular diagnostics [10, 58]. Microarray technology commonly employs an array of target-complementary oligonucleotides printed on a “chip” to which fluorescence-labelled nucleic acid from the sample is hybridized; the degree of fluorescence correlates to the abundance of the sample DNA. Because of the array format and the analog nature of fluorescence intensity, this technology is intrinsically high-throughput and somewhat quantitative, respectively. Wang et al. [55] capitalized on these advantages and combined them with the sensitivity of PCR by first using conserved, family-specific primers to amplify 1.3-kb microsporidia rRNA fragments from unextracted, FTA-filtered clinical fecal samples before hybridization to a microsporidia microarray. Multiple specific probes were then used to confer genus- and species-level hybridization profiles to the assay and to increase sensitivity by decreasing amplicon size (i.e., in a “nested” fashion). The array was able to simultaneously detect all four species of microsporidia at a sensitivity of 10^2^ spores per 100 *μ*L of fecal sample. In a survey of 20 fecal samples from AIDS patients suffering from diarrhea of unknown etiology, 12 samples were microsporidia-positive, and all but one were apparently multiply-infected. No masking effect by the more abundant species was evident, and the probe hybridization profile for each species offers a tentative assessment of infection intensity. The printing of four individual microarrays per slide increases the potential throughput of this technique.

## 3. Antigen-Based Detection Methods

Antigen-based detection methods such as the immunofluorescence assays (IFAs), ELISA, and immunoblot use antibodies from experimentally immunized animals to recognize characteristic pathogen specific antigens. IFA can be used *in situ* on fixed specimens but needs to be examined using fluorescence microscopy. Immunoblot or ELISA tests examine an homogenate of the specimen. Antibodies may be either polyclonal (i.e., purified from animal sera and directed against various epitopes of the protein, and possibly containing other, nonspecific antibodies which can increase background signal) or monoclonal (purified from cell culture supernatants). 

A number of monoclonal and polyclonal antibodies against human-infecting microsporidia including *Ent*. *bieneusi*, *Enc*. *cuniculi*, *Enc*. *hellem*, and *Enc*. *intestinalis* have been developed [59–65]. Most often these antibodies have been directed against the spore wall or polar tube of the Microsporidia. Some of these antibodies have demonstrated cross-reactivity among various species of microsporidia by IFA or immunoblot. While some investigators have reported IFA tests that had an equal sensitivity to a reference PCR (e.g., [64]), the majority of investigators believe that IFA tests are less sensitive than PCR based methods. In any case, the specificity and sensitivity depend to a great extent on the antibody itself and the care with which the various steps (e.g. fixation, blocking, and washes) are executed. Antibody-based detection is best used as a supplement to conventional histological techniques, and in difficult cases nucleotide-based detection should be utilized as well.

## 4. Antibody-Based Detection Methods

Serologic tests such as the enzyme-linked immunosorbent assay (ELISA), immunoblot, and agglutination-based tests [66, 67] which can detect circulating antibody are not currently recommended for diagnostic purposes due to variable expression of antibodies in immunocompromised patients, the inability to discriminate between acute and past infections [68], the high prevalence of anti-microsporidian antibodies in apparently healthy, immunocompetent populations [6, 7], and cross-reactivity of antibodies between different species. However, these serologic analyses may be useful to diagnose subclinical infections in prospective transplant donors or patients who may be at risk for reactivation of infection due to impending immune compromise (discussed in [8]).

## 5. Detection of Microsporidia from Environmental Samples

Because many species of microsporidia are enteric pathogens in humans and animals and are transmitted as environmentally resistant spores [69], it is likely that waterborne transmission of these parasites occurs. Human-pathogenic microsporidia have been detected in surface water, groundwater, and tertiary agricultural effluent [23, 70–75], which poses a contamination risk to drinking, recreational, and agricultural water supplies. Indeed, in 1999 there was a confirmed waterborne outbreak of microsporidiosis that affected both immune-compromised and immune-competent individuals [76]. As a result of such studies, the U.S. Environmental Protection Agency included the microsporidia on its two most recent Candidate Contaminant Lists (CCL-1 and -2) in 1998 and 2005, respectively. The CCL-2 currently consists of eight other candidate microbiological agents and 42 chemical agents which are known or anticipated to be present in public water systems and which may require regulation under the Safe Drinking Water Act.

Waterborne protozoa are usually detected from large-volume water samples by filter-based or centrifugal concentration followed by purification and molecular or microscopic identification of the organism from the concentrated material [77]. Currently, methods for the enrichment of microsporidia in water samples have not been standardized, but relatively expedient concentration of spores has been achieved by continuous flow centrifugation (CFC) [78] or continuous separation channel centrifugation [79]. In the case of water samples, purification of spores by immunomagnetic separation (IMS), which utilizes pathogen-specific antibody-coated beads prior to detection by real-time PCR, was shown to be 78%–90% sensitive for seeded spores in ultrapure water [78], although the paucity of commercially available anti-microsporidia antibodies currently limits the accessibility of this approach. In all cases requiring detection of microsporidia from turbid samples such as feces-impacted or otherwise turbid environmental water, the small size of human-pathogenic microsporidia impedes detection sensitivity, as it necessitates a reduction in filter pore size which increases membrane-fouling, thus effectively decreasing the volume of water that can be filtered [78]. In the case of feces or heavily feces-impacted wastewater, a similar problem arises in that although smaller, filtered, or unfiltered volumes may be analyzed for the sake of convenience, and to minimize the effect of PCR inhibitors usually present therein [26], such small volumes may not be representative of the entire sample or may be inadequate for detection of low-intensity contaminations (discussed in [80]). Nonetheless, detection limits of 10^2^ to 10^3^ spores per milliliter of feces or wastewater were achieved by sucrose-flotation purification followed by DNA extraction using commercial kits and PCR [80], a significant improvement over previously reported methods even for less turbid samples [71, 81].

While waterborne microsporidia likely pose the greater environmental threat, nonaquatic dispersal of microsporidia is also a public health concern. Spores have been identified on fresh produce in Poland such as berries and other fruits, sprouts, and green-leaf vegetables [82], perhaps as a consequence of microsporidial contamination of agricultural irrigation waters [74] or sewage-sludge end products used as fertilizer [83, 84]. Three species of Encephalitozoonidae were detected by FISH at levels likely to be infective for humans. In urban settings in Europe and North America, human-infecting microsporidia have been identified from pigeon fecal droppings [83–86]. The genotype of one isolate was found to match that of a previously reported human-infecting isolate [86], and in one study, 11% of pigeon fecal samples were found to be *Ent. bieneusi*-positive [85]. Graczyk et al. [83, 84] estimated that a person could inhale 10^3^ viable, aerosolized spores in 30 minutes of occupational or incidental exposure to heavily pigeon excrement-contaminated surfaces. In addition, Mathis et al. [87] demonstrated *Ent*. *bieneusi* in feces of farm dogs and cats; diagnostic PCR suggested that the strains are closely related to human isolates. These findings support the notion that human microsporidiosis is a zoonotic disease [69, 88, 89] and highlight the utility of molecular methods to identify new sources of risk to human health.

## 6. Conclusion

The potential of molecular diagnostics and particularly nucleic acid-based diagnostics to exceed traditional methods in terms of sensitivity, specificity, speed, and reproducibility has already achieved proof-of-concept for other pathogens [10, 11]. Indeed, this has been demonstrated for the microsporidia in molecular detection studies that processed corresponding specimens for light microscopy (e.g., [24, 51]). Although a blinded, multicenter evaluation of detection methods for the microsporidia conducted in 1998 (Rinder et al. [90]) revealed only a modest sensitivity advantage of PCR (89%) over light microscopy (80%), the greatest differences were seen between individual laboratories. Thus it is likely that as the molecular diagnostic methods are perfected over time and clinical diagnostic technicians become accustomed to them, their advantages will become more apparent. While the costs of such technology and requisite training of staff may seem initially prohibitive, the embedded costs of delayed and nonspecific or incorrect diagnosis to both patients and health care systems should be considered (see [11]).

In summary, molecular detection methods for the microsporidia described herein are potentially more sensitive, specific, and depend less on the subjectivity of the observer than traditional microscopy-based methods. Additionally, sophisticated nucleotide-based methods such as real-time PCR and oligonucleotide microarrays are intrinsically higher-throughput and quantitative, enabling simultaneous analysis of specimens for multiple pathogens as well as a tentative assessment of infection intensity. Although the time-to-result and reproducibility in clinical diagnostic settings have yet to be evaluated, a modest learning curve should be expected considering the “emerging” nature of these pathogens (see [91]). Looking ahead as more physiological insight into these pathogens is afforded by recent genomic sequencing projects [30, 31], these technologies may even be adapted as necessary to applications such as strain-genotyping and drug sensitivity-profiling.

## Figures and Tables

**Table 1:** Diagnostic Primers for the Microsporidia.

Species amplified	Sequence (5′ to 3′)	Primer /probe*name	Annealing temp. in PCR/*melting temp.#* (°C)	Amplicon size, base pairs	Reference(s); notes
Primers used in PCR					

*Anncaliia (Brachiola) algerae *	ACTCCGGTAACGTGTGATGTG	NALGf2	55	180	[37]
	TACAAAGCATGATCCCAGTCT	NALGR1			

*Anncaliia (Brachiola) algerae *	GCCGTTTCCGAAGTTGG	NAGf	50	192	[38]
	ATATCGACGGGACTCTCACC	NAG178r			

Encephalitozoonidae and *Ent. bieneusi *	CACCAGGTTGATTCTGCCTGAC	PMP1 (V1)	60	Eb 250	[17]
	CCTCTCCGGAACCAAACCTG	PMP2		Ec 268	
				Ei 270	
				Eh 279**	

Encephalitozoonidae and *Ent. bieneusi *	TGAATG(G/T)GTCCCTGT	MSP1	58	Eb 508	[22]
	TCACTCGCCGCTACT	MSP2A		Ec 289	
	GTTCATCGCACTACT	MSP2B		Ei 305	
	GGAATTCACACCGCCCGTC(A/G)(C/T)TAT	MSP3			
	CCAAGCTTATGCTTAAGT(C/T)(A/C)AA(A/G)GGGT	MSP4A			
	CCAAGCTTATGCTTAAGTCCAGGGAG	MSP4B			

Encephalitozoonidae and *Ent. bieneusi *	CCAGGUTGATUCTGCCUGACG	Mic3U	65/62	Eb 132	[20]
	TUACCGCGGCUGCUGGCAC	Mic421U		Ec 113	
	AAGGAGCCTGAGAGATGGCT	Mic266		Eh 134	
	CAATTGCTTCACCCTAAGGTC	Eb379		Ei 128	
	GACCCCTTTGCACTCGCACAC	Ec378			
	TGCCCTCCAGTAAATCACAAC	Eh410			
	CCTCCAATCAATCTCGACTC	Ei395			

Encephalitozoonidae and *Ent. bieneusi *	CACCAGGTTGATTCTGCC	C1 (V1)	56	Eb 1170	[18]
	GTGACGGGCGGTGTCTAC	C2		Ec 1190	
				Eh 1205	
				Ei 1186***	

Encephalitozoonidae	TGCAGTTAAAATGTCCGTAGT	int530f	40	1000	Didier et al., [39]
	TTTCACTCGCCGCTACTCAG	int580r			

*Enc. intestinalis*	CACCAGGTTGATTCTGCCTGAC	V1	58	375	[40]
	CTCGCTCCTTTACACTCGAA	Si500			

*Enc. intestinalis*	GGGGGTAGGAGTGTTTTTG	3	65	930	Schuitema et al., 1993, [41]
	CAGCAGGCTCCCTCGCCATC	3			

*Enc*. *intestinalis *	TTTCGAGTGTAAGGAGTCGA	SINTF1	55	520	[42, 43]
	CCGTCCTCGTTCTCCTGCCCG	SINTR			

*Enc. cuniculi*	ATGAGAAGTGATGTGTGTGCG	ECUNF	55	549	[43, 44]
	TGCCATGCACTCACAGGCATC	ECUNR			

*Enc. hellem*	TGAGAAGTAAGATGTTTAGCA	EHELF	55	547	[44]
	GTAAAAAGACTCTCACACTCA	EHELR			

*Ent. bieneusi*	GAAACTTGTCCACTCCTTACG	EBIEF1	55	607	[42]
	CCATGCACCACTCCTGCCATT	EBIER1			
*Ent. bieneusi*	CACCAGGTTGATTCTGCCTGAC	V1	48	353	[45, 46]
	ACTCAGGTGTTATACTCACGTC	EB450			

*Ent. bieneusi*	CACCAGGTTGATTCTGCCTGAC	V1	54	446	[24, 47]
	CAGCATCCACCATAGACAC	Mic3			

*Ent. bieneusi*	TCAGTTTTGGGTGTGGTATCGG	Eb.gc	49	210	[48]
	GCTACCCATACACACATCATTC	Eb.gt			

*Ent. bieneusi*	GCCTGACGTAGATGCTAGTC	2	55	1265	[49]
	ATGGTTCTCCAACTGAAACC	2			

*Vittaforma corneae*	TGAGACGTGAAGATGAGTATC	NCORF1	55	375	Pieniazek NJ and Visvesvara GS personal communication
	TCCCTGCCCACTGTCTCCAAT	NCORR1			

Primers and probes used in hybridization					

*Encephalitozoon spp.*	CAGGTTGATTCTGCCTGACG	FP	63		Notermans et al., [50]
	ATCTCTCAGGCTCCCTCTCC	RP			

*Enc. hellem*	ACT CTCACA CTC ACT TCA G	*HEL878F*	54		[51]

*Ent. bieneusi*	CGGTGGTGTGTGTAGGCGTGAGAGTGTATC	*probe*	55		[52]
			(hybridization)		

Primers and probes used in RT-PCR or fluorogenic PCR					

*EncephP1 *	CCC TGT CCT TTG TAC ACA CCG CCC	*EncephP1 *	68		[53]
*EcunF1*	TCC TAG TAA TAG CGG CTG ACG AA	EcunF1	59		
*EcunR2 *	ACT CAG GAC TCA GAC CTT CCG A	EcunR2	59		
*EhelF1 *	GAA TGA TTG AAC AAG TTA TTT TGA ATG TG	EhelF1	59		
*EhelR2 *	AAC ACG AAA GAC TCA GAC CTC TCA	EhelR2	58		
*EintF1 *	AAT TCC TAG TAA TAA CGA TTG AAC AAG TTG	EintF1	59		
*EintR2 *	ACG AAG GAC TCA GAC CTT CCA A	EintR2	59		

*Ent. bieneusi *	CGCTGTAGTTCCTGCAGTAAACTATGCC	FEB1	65	102	[33, 34]
	CTTGCGAGCGTACTATCCCCAGAG	REB1			
	ACGTGGGCGGGAGAAATCTTTAGTGTTCGGG	*probe*			

*Enc. intestinalis *	GCAAGGGAGGAATGGAACAGAACAG	FEI1	65	127	[33]
	TTCAGAAGCCCATTACACAGC	REI1			
	CGGGCGGCACGCGCACTACGATA	*probe*			

*Ent. bieneusi*	TGTGTAGGCGTGAGAGTGTATCTG	EbITS-89F	60	103	[54]
	CATCCAACCATCACGTACCAATC	EbITS-191R			
	CACTGCACCCACATCCCTCACCCTT	*EbITS-114revT*			
*Encephalitozoon spp.*	CACCAGGTTGATTCTGCCTGAC	MSP1F	60		
	CTAGTTAGGCCATTACCCTAACTACCA	Eint227R			
	CTATCACTGAGCCGTCC	*Eint82Trev*			

*Encephalitozoon *	GTCCGT TAT GCC CTG AGA T		60	268	
	ACA GCA GCC ATG TTA CGACT				
	GCC CGT CGC TATCTA AGA TGA CGC A	*probe 1*			
	TGG ACG AAG ATT GGA AGGTCT GAG TC	*probe 2*			

Primers and probes used in oligonucleotide microarray					

microsporidia-generic	GATTCTGCCTGACGTGGATGCTATT	* Msp1 *	58		[55]
	ATTCCGGAGAGGGAGCCTGAGAGAT	* Msp2 *	61		
	ATTGACGGAAGGACACTACCAGGA	* Msp3 *	57		
	GTGCGGCTTAATTTGACTCAACGCG	* Msp4 *	58		

*Ent. bieneusi*	ACGGCTCAGTAATGTTGCGGTAATT	* Eb1 *	56		
	CCTATCAGCTTGTTGGTAGTGTAAA	* Eb2 *	54		
	TCATGAGACGTGAGTATAAGACCTG	* Eb3 *	56		
	ATCGAATACGTGAGAATGGCAGGAGT	* Eb4 *	58		
	CTAAAAGCGGAGAATAAGGCGCAAC	* Eb5 *	58		
	CGTTGTTCAATAGCGATGAGTTTGC	* Eb6 *	56		
	GGTGAAACTTAAAGCGAAATTGACGG	* Eb7 *	56		
	AGCCTGTGTGTGAGAATACGTGG	* Eb8 *	56		

*Encephalitozoon *	ACGGCTCAGTGATAGTACGATGATT	* Ence1 *	56		
	TATCAGCTGGTAGTTAGGGTAATGG	* Ence2 *	56		
	GAGTGAAACTTGAAGAGATTGACGG	* Ence3 *	56		

*Enc. cuniculi *	TGTGGGGTTGGCAAGTAAGTTGTGG	* Ec1 *	59		
	ATGAGAAGTGATGTGTGTGCGAGTG	* Ec2 *	58		
	GCCTGTGAGTGCATGGCATGAG	* Ec3 *	59		
	GGGAAACTGCAGATAGTGGTCTGC	* Ec4 *	59		
	GGATGTAGTGATGTGTGTGGCAGAG	* Ec5 *	59		
	CTGGACGGGACAGTGTGTGTTGT	* Ec6 *	59		

*Enc. hellem *	TAAGTTCTGGGGGTGGTAGTTTGTA	* Eh1 *	56		
	GCGGTTATGAGAAGTAAGATGTTTAGCA	* Eh2 *	57		
	CTGAAGTGAGTGTGAGAGTGTTTTTAC	* Eh3 *	57		
	ATTGGGAGCCTGGATGTAACTGTGG	* Eh4 *	59		
	GGATGTAGTTTTATTGTAGCAGAGG	* Eh5 *	54		
	TGGACGGGACTGTTTTAGTGTTGTC	* Eh6 *	58		
*Enc. intestinalis *	TTGACACGAGCCAAGTAAGTTGTAG	* Ei1 *	56		
	TTTCGAGTGTAAAGGAGTCGAGATTGA	* Ei2 *	57		
	GGCAGGAGAACGAGGACGGGAT	* Ei3 *	60		
	CAGGTAGGGGGCTAGGAGTGTTTTT	* Ei4 *	59		
	TATGTCCTGATGTGGATGTAAGAGG	* Ei5 *	56		
	TGGACGGGACTATATAGTGTTGTG	* Ei6 *	56		

microsporidia-generic	TTTMMAACGGCCATGCACCAC	MspR3	52	var.	Used to label initial PCR products

* Italics indicate hybridization probe. ** PstI and HaeIII restriction analysis differentiates these amplicons. *** HindIII and HinfI restriction analysis differentiates these amplicons. ^*#*^ Italics indicate melting temperature.

**Table 2:** Primers for the identification and sequencing of microsporidian rRNA^1^ Genes.

ss^2^18f^3^	CACCAGGTTGATTCTGCC
ss18sf	GTTGATTCTGCCTGACGT
ss350f	CCAAGGA(T/C)GGCAGCAGGCGCGAAA
ss350r	TTTCGCGCCTGCTGCC(G/A)TCCTTG
ss530f	GTGCCAGC(C/A)GCCGCGG
ss530r	CCGCGG(T/G)GCTGGCAC
ss1047r	AACGGCCATGCACCAC
ss1061f	GGTGGTGCATGGCCG
ss1492r	GGTTACCTTGTTACGACTT (Universal primer)
ss1537	TTATGATCCTGCTAATGGTTC
ls212r1	GTT(G/A)GTTTCTTTTCCTC
ls212r2	AATCC(G/A/T/C)(G/A)GTT(G/A)GTTTCTTTTCCTC
ls580r	GGTCCGTGTTTCAAGACGG

1- Primers 18f and 1492r amplify most of the small subunit rRNA of the microsporidia. Primes 530f and 212r1 or 212r2 are used to amplify the small subunit rRNA and the ITS region. The remaining primers are used to sequence, with overlap, the forward and reverse strands of the entire small subunit rRNA and ITS region. ls580r amplifies a variable region of the 5′ end of the large subunit rRNA gene of many microsporidia (e.g., Nosema and Vairimorpha) but it does not work on all microsporidia. ss1537 allows sequencing closer to the 3′ end of the small subunit rRNA of many but not all microsporidia. ss350f and ss350r may not be needed for sequencing reactions if 18f and 530r provide sufficient overlap to obtain clear sequence data. 2- ss: denotes primers in the small subunit rRNA gene, **ls: denotes primers in the large subunit rRNA gene, **f: forward primer (positive strand), **r: reverse primer (negative strand). 3- Similar to V1 primer. Adapted from [12].
